# #nowplaying Madonna: a large-scale evaluation on estimating similarities between music artists and between movies from microblogs

**DOI:** 10.1007/s10791-012-9187-y

**Published:** 2012-03-06

**Authors:** Markus Schedl

**Affiliations:** Department of Computational Perception, Johannes Kepler University, Altenberger Straße 69, 4040 Linz, Austria

**Keywords:** Social media mining, Microblog analysis, Vector space model, Term weighting, Information extraction, Evaluation

## Abstract

Different term weighting techniques such as $TF\cdot IDF$ or *BM25* have been used intensely for manifold text-based information retrieval tasks. Their use for modeling term profiles for named entities and subsequent calculation of similarities between these named entities have been studied to a much smaller extent. The recent trend of microblogging made available massive amounts of information about almost every topic around the world. Therefore, microblogs represent a valuable source for text-based named entity modeling. In this paper, we present a systematic and comprehensive evaluation of different *term weighting measures*, *normalization techniques*, *query schemes*, *index term sets*, and *similarity functions* for the task of inferring similarities between named entities, based on data extracted from *microblog posts*. We analyze several thousand combinations of choices for the above mentioned dimensions, which influence the similarity calculation process, and we investigate in which way they impact the quality of the similarity estimates. Evaluation is performed using three real-world data sets: two collections of microblogs related to music artists and one related to movies. For the music collections, we present results of *genre classification experiments* using as benchmark genre information from allmusic.com. For the movie collection, we present results of *multi-class classification experiments* using as benchmark categories from IMDb. We show that microblogs can indeed be exploited to model named entity similarity with remarkable accuracy, provided the correct settings for the analyzed aspects are used. We further compare the results to those obtained when using Web pages as data source.

## Introduction

Microblogging has encountered a tremendous popularity gain during the past couple of years. Today’s most popular microblogging service Twitter
1 has more than 100 million registered users (Yarow 2011). Millions of users post “tweets” that reveal what they are doing, what is on their mind, or what is currently important for them. According to Evans (2011), the number of tweets per day surpassed 50 millions in early 2010. Twitter thus represents a rich data source for text-based information extraction (IE) and information retrieval (IR).

In classical text-IR, term weighting techniques such as $TF\cdot IDF$ and *BM25* are typically used in combination with a similarity function to estimate the relevance of a set of documents to a query. In IE the same techniques (term weighting and similarity calculation) can be used to model term profiles for named entities and compute pairwise similarity scores between these entities. Such similarity measures are vital for various applications, in particular, in the domain of multimedia retrieval. For example, in music information retrieval elaborating musical similarity measures that are capable of capturing aspects that relate to real, perceived similarity is one of the main challenges as it enables a wealth of intelligent music applications. Examples are systems to automatically generate playlists (Aucouturier and Pachet 2002; Pohle et al. 2007), music recommender systems (Celma 2008; Zadel and Fujinaga 2004), music information systems (Schedl 2008), semantic music search engines (Knees et al. 2007), and intelligent user interfaces (Knees et al. 2007; Pampalk and Goto 2007) to access music collections by means more sophisticated than the textual browsing facilities (artist-album-track hierarchy) traditionally offered.

Various approaches to model the term vector space (Salton et al. 1975) on the Web have been proposed throughout the last years, e.g., Debole and Sebastiani (2003), Lan et al. (2005), Salton and Buckley (1988), Schedl et al. (2011), Whitman and Lawrence (2002). Microblogs, in contrast, have been studied to a much smaller extent, although using this data source for the purpose of similarity estimation between entities offers several advantages over the use of Web pages. First, microblog posts are shorter and typically more precise than Web pages, the former reducing computational complexity, the latter potentially offering more accurate results. Second, due to the instantaneous nature of microblogs, text-based similarity estimation approaches leveraging this kind of data are better capable of incorporating breaking news and offering a more up-to-date view on events related to the investigated domains, such as information on album releases or latest gossip about musicians or actors.

Addressing the lack of literature on modeling named entities via term vectors on the microblogosphere and thoroughly investigating different aspects of the models, the work at hand is the first aiming to answer the following research questions. First, we would like to assess if microblog data gathered over several months are capable of reflecting similarities between named entities from two domains, namely music artists and movies. We chose these two domains because accurate similarity measures are of particular importance in these contexts, which is underlined by the recent popularity and developments of recommender systems for music and movies, such as those offered by last.fm and Netflix, cf. Celma (2008), Koren (2009). The second important question that is addressed in this work is how to model similarities between the entities of interest. There exists a large number of possibilities to construct term vectors from texts/microblogs related to the named entities under consideration (in regard to term selection, term weighting, or normalization, for example). The corresponding algorithmic choices, together with the actual similarity measure employed, have a great impact on the accuracy of the similarity estimates between the music or movie entities. The objective of this work is hence to identify well-performing combinations of these choices and to derive general rules for modeling similarities between named entities from microblogs. Performance is measured by an evaluation approach resembling (Sanderson and Zobel 2005). More precisely, Mean Average Precision (MAP) scores are computed on genre labels predicted by a k-Nearest Neighbor (kNN) classifier. To reduce the computational complexity of evaluating the otherwise enormous set of different algorithmic combinations, results are first computed on a smaller set and only combinations statistically insignificantly different from the top-performing combination will be assessed on the larger data sets.

The work at hand was inspired by Zobel and Moffat (1998), where the authors thoroughly evaluate various choices related to constructing text feature vectors for IR purposes, e.g., term frequency (*TF*), term weights (*IDF*), and normalization approaches. They analyze the influence of these decisions on retrieval behavior. Similarly, a systematic large-scale study (in terms of single evaluation experiments and factors analyzed) on the influence of a multitude of decisions on similarity estimation, using real-world data collections, is presented here. To this end, we investigate several thousand combinations of the following single aspects:

- query scheme
- index term set
- term frequency
- inverse document frequency
- normalization with respect to document length
- similarity function

The *term frequency*  *r*_*d*,*t*_ of a term *t* in a document *d* estimates the importance *t* has for document *d* (representing the named entity under consideration). The *inverse document frequency*  *w*_*t*_ estimates the overall importance of term *t* in the whole corpus and is commonly used to weight the *r*_*d*,*t*_ factor, i.e., downweight terms that are important for many documents and hence less discriminative for *d*. We further assess the impact of *normalization* with respect to document length. Moreover, different *similarity functions*  $S_{d_{1}, d_{2}}$ to estimate the proximity between the term vectors of two named entities’ documents *d*_1_ and *d*_2_ are examined.

The remainder of this article is organized as follows. Section 2 outlines the context of this work by conducting a literature review on text-based similarity measurement and microblog mining. Section 3 then describes all aspects we analyzed to model the named entity similarity space on the microblogosphere. The core part of this contribution can be found in Section 4, where details on the experiments are given and results are presented and discussed. Finally, conclusions are drawn in Section 5.

## Related work

Related work basically falls into two categories: text-based similarity measurement and microblog mining. Whereas the former has a long tradition, ranging back several decades, the latter is a rather young research field.

### Text-based similarity measures

There exists a wide range of literature on modeling text documents according to the bag-of-words principle using vector space representations, e.g., Baeza-Yates and Ribeiro-Neto (2011), Luhn (1957), Salton et al. (1975). Since elaborating on all publications related to the discipline of text-IR is out of this article’s scope, we restrict ourselves to point to some work dealing with text-IR in the context of multimedia retrieval on the Web, as this context is closely related to the sets of named entities we use in the evaluation experiments.

Text data in the multimedia domain generally constitutes *context information* or *contextual data*, opposed to content-based features directly extracted from the media items. Deriving term feature vectors from Web pages for the purpose of music artist similarity calculation was first undertaken in Cohen and Fan (2000). Cohen and Fan automatically extract lists of artist names from Web pages, which are found by querying Web search engines. The resulting pages are then parsed according to their DOM tree, and all plain text content with minimum length of 250 characters is further analyzed for occurrences of entity names. Term vectors of co-occurring artist names are then used for artist recommendation. Using artist names to build term vector representations, whose term weights are computed as co-occurrence scores, is an approach also followed later in Schedl et al. (2005), Zadel and Fujinaga (2004). In contrast to Cohen and Fan’s approach, the authors of Schedl et al. (2005), Zadel and Fujinaga (2004) derive the term weights from search engine’s page count estimates and suggest their method for artist recommendation.

Automatically querying a Web search engine to determine pages related to a specific topic is a common and intuitive task, which is therefore frequently performed for data acquisition in IE research. Examples in the music domain can be found in Geleijnse and Korst (2006), Whitman and Lawrence (2002), whereas Cimiano et al. (2004), Cimiano and Staab (2004), Knees et al. (2007) apply this technique in a more general context.

Building term feature vectors from term sets other than artist names is performed in Whitman and Lawrence (2002), where Whitman and Lawrence extract different term sets (unigrams, bigrams, noun phrases, artist names, and adjectives) from up to 50 artist-related Web pages obtained via a search engine. After downloading the pages, the authors apply parsers and a part-of-speech (POS) tagger (Brill 1992) to assign each word to its suited test set(s). An individual term profile for each artist is then created by employing a version of the $TF\cdot IDF$ measure. The overlap between the term profiles of two artists, i.e., the sum of weights of all terms that occur in both term profiles, is then used as an estimate for their similarity.

Extending the work presented in Whitman and Lawrence (2002), Baumann and Hummel (2003) introduce filters to prune the set of retrieved Web pages. First, they remove all Web pages with a size of more than 40 kilobytes (after parsing). They also try to filter out advertisements by ignoring text in table cells comprising more than 60 characters, but not forming a correct sentence. Finally, Baumann and Hummel perform keyword spotting in the URL, the title, and the first text part of each page. Each occurrence of the initial query parts (artist name, “music”, and “review”) contributes to a page score. Pages that score too low are filtered out.

Knees et al.’s (2004) approach is similar to Whitman and Lawrence (2002). Unlike Whitman and Lawrence who experiment with different term sets, Knees et al. use only one list of unigrams. For each artist, a weighted term profile is created by applying a $TF\cdot IDF$ variant. Calculating the similarity between the term profiles of two artists is then performed using the cosine similarity. Knees et al. evaluate their approach in a genre classification setting using as classifiers k-Nearest Neighbor (kNN) and Support Vector Machines (SVM) (Vapnik 1995).

Other approaches derive term profiles from more specific Web resources. In Celma et al. (2006), for example, the authors propose a music search engine that crawls audio blogs via RSS feeds and calculates $TF\cdot IDF$ features. Hu et al. (2005) extract *TF*-based features from music reviews gathered from Epinions.com.^2^ In Schedl (2010) the author extracts user posts associated with music artists from the microblogging service Twitter
3 and models term profiles using term lists specific to the music domain.

In the work reported on so far, the authors usually select a specific variant of the $TF\cdot IDF$ term weighting measure and apply it to documents retrieved for the entity under consideration. The individual choices involved in selecting a specific $TF\cdot IDF$ variant and similarity function, however, do not seem to be the result of detailed assessments. They rather resemble common variants that are known to yield good results in IR tasks. Whether these variants are also suited to describe named entities via term profiles and subsequently estimate similarities between them is seldom assessed comprehensively in the literature. Sebastiani (2002) presents a review of different approaches to text categorization from a machine learning perspective, focusing on term selection techniques. Salton and Buckley (1988) investigate different approaches to term weighting and similarity measurement for text retrieval. Closest to the work at hand is certainly Zobel and Moffat’s thorough study on various choices in modeling term profiles (Zobel and Moffat 1998). In particular, term weights for queries and documents as well as similarity functions are analyzed. However, Zobel and Moffat aim at determining good algorithmic choices for the purpose of document retrieval, i.e., retrieving relevant documents for a given query. We are, in contrast, interested in similarity measurement between two documents that represent named entities. Therefore, this article presents the first comprehensive study on named entity similarity estimation on the microblogosphere.

### Microblog mining

With the advent of microblogging a huge, albeit noisy data source became available. Literature dealing with microblogs can be broadly categorized into works that study human factors or properties of the Twittersphere and works that exploit microblogs for information extraction and retrieval tasks.

As for the former, Teevan et al. (2011) analyze query logs to uncover differences in search behavior between users of classical Web search engines and users looking for information in microblogs. They found that Twitter queries are shorter and more popular than bing
4 queries on average. Furthermore, microblogs are more often sought for people, opinions, and breaking news. In terms of query formulation, reissuing the same query can be more frequently observed in microblog search. In Web search, by contrast, modifying and extending a query is very popular.

Java et al. (2007) study network properties of the microblogosphere as well as geographical distributions and intentions of Twitter users. The authors report that Twitter is most popular in North America, Europe, and Asia (Japan), and that same language is an important factor for cross-connections (“followers” and “friends”) over continents. Employing the *HITS* algorithm (Kleinberg 1999) on the network of “friend”-relations, Java et al. further derived user intentions from structural properties. They identified the following categories: information sharing, information seeking, and friendship-wise relationships. Analyzing the content of Twitter posts, the authors distilled the following intentions: daily chatter, conversations, sharing information/URLs, and reporting news.

In a recent study, Kwak et al. (2010) perform a topological analysis of the Twitter network. The authors report a low level of reciprocity, i.e., only 22% of the connections between users are bidirectional. The average path length was found to be only four, which is surprisingly small for a network the size of the Twittersphere and considering the directional network structure. Moreover, a moderate level of homophily, i.e., a higher likelihood for connections between similar people than between dissimilar people, was discovered when measuring similarity in terms of geographic location and user popularity. In addition, Kwak et al.’s study indicates that information diffusion after the first retweet is very fast.

Work related to content mining of microblogs includes the following: Cheng et al. propose a method to localize Twitter users based on spatial cues (“local” words) extracted from their tweets’ content (Cheng et al. 2010). To this end, in a first step several classifiers are trained to identify words with a strong geospatial meaning. In order to deal with the sparsity in the distribution of these cues, different smoothing approaches, e.g., taking into account neighboring cities when constructing the term representation of a city, are applied subsequently. In an experiment conducted on a set of tweets posted within the USA, Cheng et al.’s approach placed more than a half of the users within a 100-mile-radius of their correct location.

Making use of the fact that tweets are a good source for up-to-date information and breaking news, Dong et al. (2010) propose an approach to identify fresh URLs in Twitter posts. To this end, the authors investigate content-based features extracted from the tweets, an authority score computed for each user, and Twitter-specific statistical features, such as number of retweets or number of users that replied to a message containing a tiny URL. They show that these features can be used to improve both recency ranking and relevance ranking in real-time Web search. Another work that aims at improving ranking can be found in Duan et al. (2010). Duan et al. propose a novel ranking strategy for tweet retrieval. To this end, they investigate different feature sets, including content-based features, Twitter-specific features, and authority scores of users (followers, retweeters, mentioners). Using a learning to rank algorithm, the authors found that the best-performing features are authority scores, length of a tweet, and whether the tweet contains a URL.

An approach to classifying tweets can be found in Sriram et al. (2010). Sriram et al. describe each tweet by an eight-dimensional feature vector comprising the author of the post and seven binary attributes indicating, for example, occurrence of slang words, currency and percentage signs, or the use of capitalization and repeated characters. Sriram et al.’s feature set outperformed the standard bag-of-words approach using a Naïve Bayes classifier to categorize tweets into the five classes news, events, opinions, deals, and private messages.

Armentano et al. (2011) present a recommender system that suggests potentially interesting users to follow based on the similarity between tweets posted by the seed user and tweets posted by a set of candidate users. To this end, the authors create and investigate different user profiles, for example, modeling the seed user via term frequencies of his/her aggregate posts or of all of his/her followees. Related to Armentano et al.’s work, Weng et al. aim at identifying influential twitterers for a given topic (Weng et al. 2010). To this end, they apply *Latent Dirichlet Allocation* (LDA) (Blei et al. 2003) to their corpus of tweets. Subsequently, topical similarity between twitterers is computed as the Jensen–Shannon divergence between the distribution of the latent topics of the respective users. Further taking into account the link structure, Weng et al. propose a ranking function for influential twitterers in each topic. Similar to Armentano et al. (2011), Weng et al. evaluate their approach in a recommendation setting.

Microblogs have also been exploited for the purpose of event and trend detection. Sakaki et al. propose semantic analysis of tweets to detect earthquakes in Japan in real-time (Sakaki et al. 2010). A more general approach to automatically detect events and summarize trends by analyzing tweets is presented by Sharifi et al. (2010). Another work on trend detection is Schedl (2011), where Schedl exploits tweets for spatio-temporal popularity estimation of music artists. Sankaranarayanan et al. aim at capturing tweets that report on breaking news (Sankaranarayanan et al. 2009). They cluster the identified tweets according to their $TF\cdot IDF$ weights and cosine similarity. Furthermore, each cluster is assigned a set of geographic locations using both spatial clues in the tweets themselves and explicit location information as indicated by the twitterers.

## Modeling the microblog term vector space

Resembling the large-scale experiments conducted in Zobel and Moffat (1998), our analysis is guided by the question whether specific algorithmic choices perform consistently and considerably better or worse than others. Performance is measured via classification tasks among term vector representations of tweets, cf. Sect. 4. Our goal is, hence, to derive guidelines for favoring or avoiding specific algorithmic variants when the task is similarity estimation between named entities and the corpus comprises microblogs. The assessed aspects for modeling named entities based on microblogs are detailed in the following (Table 1).

**Table 1:** Denominations used in term weighting functions and similarity measures

${{\mathcal D}}$	Set of documents
*N*	Number of documents
*f*_*d*,*t*_	Number of occurrences of term *t* in document *d*
*f*_*t*_	Number of documents containing term *t*
*F*_*t*_	Total number of occurrences of *t* in the collection
${{\mathcal T}_d}$	Set of distinct terms in document *d*
*f*_*d*_^*m*^	Largest *f*_*d*,*t*_ of all terms *t* in *d*
*f* ^*m*^	Largest *f*_*t*_ in the collection
*r*_*d*,*t*_	Term frequency (cf. Table 5)
*w*_*t*_	Inverse document frequency (cf. Table 6)
*W*_*d*_	Document length of *d*

### Query scheme

We decided to assess two different schemes to query Twitter as previous work on Web-based IE (Schedl et al. 2005; Whitman and Lawrence 2002) has shown that adding domain-specific key terms to a search request generally improves the quality of feature vectors in terms of similarity-based classification accuracy. In Web-based music information research, for example, common terms used as additional key words are “music review” or “music genre style”. Taking into account the 140-character-limitation of tweets, we decided to include only “music” as additional query term (QS_M) for the music data sets, or we query without any additional key terms, i.e., use only the artist name (QS_A). For the movie data set, the setting QS_M refers to including the term “movie” in the query. Table 2 summarizes the two query schemes investigated.

**Table 2:** Query schemes used to retrieve music/movie-related tweets

Abbr.	Query scheme
QS_A	“artist name” / “movie name”
QS_M	“artist name”+music / “movie name”+movie

### Index term set

Earlier work in text-based music artist modeling (Turnbull et al. 2007; Hu and Downie 2007; Pampalk et al. 2005) shows that a crucial choice in defining the representation of an artist is that of the terms used to index the corresponding documents. For the work at hand, we hence investigated various term sets, which are summarized for the music and movie collections, respectively, in Tables 3 and 4. Set TS_A contains all terms found in the corpus (after casefolding, stopping, and stemming). Set TS_S is the entire term dictionary of SCOWL,^5^ which is an aggregation of several spell checker dictionaries for various English languages and dialects. Set TS_N encompasses all artist names present in the music data set. Previous work has shown that the corresponding *co-occurrence* approach to music artist similarity estimation yields remarkable results (Schedl and Knees 2008; Schedl et al. 2005). Term set TS_D is a manually created dictionary of music-related terms that resembles the one used in Pampalk et al. (2005). It contains, for example, descriptors of genre, instruments, geographic locations, epochs, moods, and musicological terms. Set TS_L represents the 250 most popular tags utilized by users of last.fm. Set TS_F comprises the aggregated data sets for the data types *musical genre*, *musical instrument*, and *emotion*, extracted from Freebase.^6^

**Table 3:** Different term sets used to index the music-related Twitter posts

Abbr./term set	Cardinality	Description
TS_A /all_terms	C224a, QS_A: 38,133 C224a, QS_M: 19,133 C3ka, QS_A: 1,489,459 C3ka, QS_M: 437,014	All terms (stemmed) that occur in the corpus of the retrieved Twitter posts
TS_S /scowl_dict	698,812	All terms that occur in the entire SCOWL dictionary
TS_N /artist_names	224/3,000	Names of the artists for which data was retrieved
TS_D /dictionary	1,398	Manually created dictionary of musically relevant terms
TS_L /last.fm_toptags	250	Overall top-ranked tags returned by last.fm’s *Tags.getTopTags* function
TS_F /freebase	3,628	Music-related terms extracted from Freebase (genres, instruments, emotions)

**Table 4:** Different term sets used to index the movie-related Twitter posts

Abbr./term set	Cardinality	Description
TS_A /all_terms	QS_A: 1,843,286/54,378 QS_M: 754,067/29,532	All terms (stemmed) that occur in the corpus of the retrieved Twitter posts
TS_S /scowl_dict	QS_A: 698,812/28,355 QS_M: 698,812/12,473	All terms that occur in the entire SCOWL dictionary
TS_D /dictionary	QS_A: 25,527/4,877 QS_M: 25,527/3,569	Dictionary of filtered IMdb key words

For the movie data set (cf. Table 4), we adapted the term sets accordingly. Sets TS_A and TS_S conceptually equal the corresponding sets used to index music-related tweets. Term set TS_D, in contrast, is a dictionary of movie-related terms, which we extracted from the “key words” provided by IMDb. Since this key word set is considerably noisy, we performed frequency-based filtering. We retained only terms that were assigned to at least 10 different movies, but to not more than 100 different movies. The former constraint effectively removes noise, the latter discards terms that are unlikely to discriminate well between different categories of movies.

To build the inverted word-level index (Zobel and Moffat 2006), we use a modified version of the open source indexer Lucene,^7^ which we extended to represent Twitter posts. The extensions will be made available through the CoMIRVA framework^8^ (Schedl et al. 2007). When creating the indexes for the different term sets, we commonly employ casefolding and stopping, e.g., Baeza-Yates and Ribeiro-Neto (2011). Stemming, in contrast, is only performed for the term sets for which it seems reasonable, i.e., for term sets TS_A and TS_S.

### TF and IDF: term weighting

The term weighting models investigated here resemble Zobel and Moffat’s (1998). We decided to extend the $TF\cdot IDF$ formulations investigated by them with *BM25*-like formulations. The assessed variants for *TF* can be found in Table 5, those for *IDF* are shown in Table 6. Table 1 contains an overview of the denominations used in the different term weighting formulations, normalization strategies, and similarity measures (Tables 7, 8).

**Table 5:** Evaluated variants to calculate the term frequency *r*_*d*,*t*_

Abbr.	Description	Formulation
TF_A	Formulation used for binary match SB = b	$${ r_{d,t} = \left\{ \begin{array}{ll} 1 & \hbox{if }\,\, t\, \in {\mathcal T}_d \\ 0 & \hbox{otherwise} \\ \end{array} \right.}$$
TF_B	Standard formulation SB = t	*r*_*d*, *t*_ = *f*_*d*,*t*_
TF_C	Logarithmic formulation	*r*_*d*,*t*_ = 1 + log _*e*_ *f*_*d*,*t*_
TF_C2	Alternative logarithmic formulation suited for *f*_*d*,*t*_ < 1	*r*_*d*,*t*_ = log _*e*_ (1 + *f*_*d*,*t*_)
TF_C3	Alternative logarithmic formulation as used in *ltc* variant	*r*_*d*,*t*_ = 1 + log _2_ *f*_*d*,*t*_
TF_D	Normalized formulation	$r_{d,t} = \frac{f_{d,t}}{f_{d}^{m}}$
TF_E	Alternative normalized formulation. Similar to Zobel and Moffat (1998) we use *K* = 0.5. SB = *n*	$r_{d,t} = K + (1-K)\cdot \frac{f_{d,t}}{f_{d}^{m}}$
TF_F	Okapi formulation, according to Robertson et al. (1995), Zobel and Moffat (1998). For *W* we use the vector space formulation, i.e., the Euclidean length	$r_{d,t} = \frac{f_{d,t}}{f_{d,t}+W_d / av_{d\in D}(W_d)}$
TF_G	Okapi BM25 formulation, according to Robertson et al. (1999)	$r_{d,t} = \frac{(k_1+1) \cdot f_{d,t}}{f_{d,t} + k_1 \cdot\left[ (1-b) + b \cdot \frac{W_d}{av_{d\in D}(W_d)} \right]}$
		*k*_1_ = 1.2, *b* = 0.75

**Table 6:** Evaluated variants to calculate the inverse document frequency *w*_*t*_

Abbr.	Description	Formulation
IDF_A	Formulation used for binary match SB = *x*	*w*_*t*_ = 1
IDF_B	Logarithmic formulation SB = *f*	$w_t = \log _e\left( 1 + \frac{N}{f_t} \right)$
IDF_B2	Logarithmic formulation used in *ltc* variant	$w_t = \log _e\left(\frac{N}{f_t} \right)$
IDF_C	Hyperbolic formulation	$w_t = \frac{1}{f_t}$
IDF_D	Normalized formulation	$w_t = \log _e\left( 1 + \frac{f_m}{f_t} \right)$
IDF_E	Another normalized formulation SB = *p*	$w_t = \log _e \frac{N - f_t}{f_t}$
	The following definitions are based on the term’s noise *n*_*t*_ and signal *s*_*t*_.	$n_t = \sum\limits_{d \in \mathcal D_t } {\left( { - \frac{{f_{d,t} }}{{F_t }}\log _2 \frac{{f_{d,t} }}{{F_t }}} \right)}$
		*s*_*t*_ = log _2_ (*F*_*t*_ − *n*_*t*_)
IDF_F	Signal	*w*_*t*_ = *s*_*t*_
IDF_G	Signal-to-noise ratio	$w_t = \frac{s_t}{n_t}$
IDF_H		$w_t = \left( {\mathop {\max n_{t^{\prime}} }\limits_{t^{\prime} \in \mathcal T} } \right) - n_t$
IDF_I	Entropy measure	$w_t = 1 - \frac{n_t}{\log _2 N}$
IDF_J	Okapi BM25 IDF formulation, according to Pérez-Iglesias et al. (2009), Robertson et al. (1999)	$w_t = \log \frac{N-f_t+0.5}{f_t+0.5}$

**Table 7:** Evaluated normalization strategies for document length

Abbr.	Description	Formulation
NORM_NO	No normalization	
NORM_SUM	Normalize sum of each virtual document’s term feature vector to 1	$\sum\limits_{t \in \mathcal T_{d}}{r_{d,t}} = 1$
NORM_MAX	Normalize maximum of each virtual document’s term feature vector to 1	$\max\limits_{t \in \mathcal T _{d}}{r_{d,t}} = 1$

**Table 8:** Evaluated similarity functions $S_{d_{1}, d_{2}}$

Abbr.	Description	Formulation
SIM_INN	Inner product	$S_{d_1,d_2} = \sum\limits_{t \in \mathcal T _{d_1,d_2}}{\left( w_{d_1,t} \cdot w_{d_2,t} \right) }$
SIM_COS	Cosine measure	$S_{d_1,d_2} = \frac{\sum_{t \in \mathcal T _{d_1,d_2}}{\left( w_{d_1,t} \cdot w_{d_2,t} \right)}}{W_{d_1} \cdot W_{d_2}}$
SIM_DIC	Dice formulation	$S_{d_1,d_2} = \frac{2\sum_{t \in \mathcal T _{d_1,d_2}} \left( w_{d_1,t} \cdot w_{d_2,t} \right) }{W_{d_1}^2 + W_{d_2}^2}$
SIM_JAC	Jaccard formulation	$S_{d_1,d_2} = \frac{\sum_{t \in \mathcal T _{d_1,d_2}} \left( w_{d_1,t} \cdot w_{d_2,t} \right) }{W_{d_1}^2 + W_{d_2}^2 - \sum_{t \in \mathcal T _{d_1,d_2}} \left( w_{d_1,t} \cdot w_{d_2,t} \right) }$
SIM_OVL	Overlap formulation	$S_{d_1,d_2} = \frac{\sum_{t \in \mathcal T _{d_1,d_2}} \left( w_{d_1,t} \cdot w_{d_2,t} \right) }{\min ( W_{d_1}^2, W_{d_2}^2 )}$
SIM_EUC	Euclidean similarity	$D_{d_1,d_2} = \sqrt{\sum\limits_{t \in \mathcal T _{d_1,d_2}} {\left( w_{d_1,t} - w_{d_2,t} \right)^2}}$
		$$S_{d_1,d_2} = \left( \max _{d_1^{\prime},d_2^{\prime}} (D_{d_1^{\prime},d_2^{\prime}}) \right) - D_{d_1,d_2}$$
SIM_JEF	Jeffrey divergence-based similarity	$S_{d_1,d_2} = \left( \max _{d_1^{\prime},d_2^{\prime}} (D_{d_1^{\prime},d_2^{\prime}}) \right) - D_{d_1,d_2}$
		$$D\left( {F,G} \right) = \sum\limits_i {\left( {f_i \log \frac{{f_i }}{{m_i }} + g_i \log \frac{{g_i}}{{m_i}}} \right)}$$
		$$m_i=\frac{f_i+g_i}{2}$$

*BM25* is an alternative term weighting scheme, used in the Okapi framework for text-based probabilistic retrieval, cf. Robertson et al. (1995, 1999). This model assumes a priori knowledge on topics from which different queries are derived. Moreover, based on information about which documents are relevant for a specific topic and which are not, the term weighting function can be tuned to the corpus under consideration. Since *BM25* is a well-established term ranking method, we included it in the experiments. However, it has to be noted that we cannot assume categorical a priori knowledge here, neither on the level of single tweets, nor on the level of named entities. On the level of tweets, manually classifying hundreds of thousands of posts would be too labor-intensive. On the named entity level, we could obviously group the entities (or more precisely, the corresponding tweets) according to a genre taxonomy and optimize *BM25* correspondingly. However, we believe that this is not justifiable for two reasons: First, for arbitrary media repositories, we cannot assume to have access to genre information. Second, using genre information would obviously bias the results of the genre classification experiments as the other term weighting measures do not incorporate such a priori knowledge. Thus, *BM25* would be unjustifiably favored. For our experiments, we therefore use a simpler *BM25* formulation as the one proposed in Robertson et al. (1999), cf. variants TF_G and IDF_J in Tables 5 and 6, respectively.

### Virtual documents and normalization

When creating a Web-based term profile that describes a named entity (a music artist or movie in our case), it is common to aggregate the Web pages associated with the entity under consideration to form a “virtual document” (Baumann and Hummel 2003; Knees et al. 2004). This procedure not only facilitates handling small or empty pages, it is also more intuitive since the item of interest is the entity under consideration, not a Web page. The study conducted in Schedl et al. (2011) further shows that calculating term weights on the level of individual Web pages before aggregating the resulting feature vector performs inferior for the task of similarity calculation than using “virtual documents”. Therefore it seems reasonable to aggregate all tweets retrieved for a named entity to one “virtual post”, in particular taking into consideration the already strong limit of Twitter posts to 140 characters.

Since the different length of two entity’s virtual documents might influence the performance of retrieval and similarity prediction tasks, e.g., Baeza-Yates and Ribeiro-Neto (2011), we evaluate several normalization methods, which are summarized in Table 7.

### Similarity function

The similarity measures analyzed are shown in Table 8. We included all measures investigated by Zobel and Moffat (1998) that can be applied to our somewhat differing usage scenario of computing similarities between two equally dimensional term feature vectors that represent two comparable named entities. In addition, Euclidean similarity (SIM_EUC) and similarity inferred from Jeffrey divergence (SIM_JEF) (Lin 1991) were included.

### Notation

To facilitate referring to a particular evaluation experiment, which is defined as a combination of the choices described above, we adopt the following scheme to denote one algorithmic setting:

- <Query Scheme>.<Index Term Set>.<Normalization>.
-    <TF>.<IDF>.<Similarity Measure>

Omitting certain components, we denote sets of algorithmic combinations: e.g., TF_C.IDF_B.SIM_COS refers to all experiments with term frequency formulation TF_C, inverse document formulation IDF_B, and the cosine similarity function, irrespective of query scheme, index term set, and document normalization.

## Evaluation

### Data sets

We performed evaluation using three data sets, covering two types of named entities that relate to two different media types: *music artists* and *movie titles*. The creation of these data sets is outlined and their properties are presented in the following.

#### Music artists

We used two data sets of music artists for evaluation. The first one, referred to as C224a, consists of 224 well-known artists and has a uniform genre distribution (14 genres,^9^ 16 artists each). It has been frequently used to evaluate Web-/text-based music information retrieval approaches.^10^

The second data set consists of 3,000 music artists, representing a large real-world collection. The data has been gathered as follows. We used last.fm’s API^11^ to extract the most popular artists for each country of the world, which we then aggregated into a single list of 201,135 unique artist names. Since last.fm’s data is prone to misspellings or other mistakes due to its collaborative, user-generated knowledge base, we cleaned the data set by matching each artist name with the database of the expert-based music information system allmusic.com,^12^ from which we also extracted genre information. Starting this matching process from the most popular artist found by last.fm and including only artist names that also occur in allmusic.com, we eventually obtained a list of 3,000 music artists. This artist set, which will be denoted C3ka in the following, is publicly available.^13^ According to allmusic.com the artists are categorized into 18 distinct genres. The distribution of the genres in C3ka is shown in Table 9. Please note that the editors of allmusic.com use the genre “Rock” to denote a widespread range of music; basically, everything from Pop to Dark Metal is classified as “Rock”. Therefore, the genre distribution is considerably unbalanced.

**Table 9:** Genre distribution of music artist set C3ka

Genre	Artists	Share (%)
Avantgarde	8	0.267
Blues	11	0.367
Celtic	5	0.167
Classical	42	1.400
Country	24	0.800
Easy listening	6	0.200
Electronica	149	4.967
Folk	24	0.800
Gospel	23	0.767
Jazz	106	3.533
Latin	91	3.033
Newage	18	0.600
Rap	203	6.767
Reggae	29	0.967
RnB	101	3.367
Rock	2,031	67.700
Vocal	30	1.000
World	99	3.300

#### Movies

The second data set consists of 1,008 distinct movie titles extracted from IMDb (Jass 2003). For 25 movie genres, we gathered the 50 top-ranked movies. We further added the overall 50 top-ranked movies of each decade, from the 1910s to the 2010s. This adds a further 11 categories. Please note that some movies occur in the top-ranked list for more than one genre, hence the total number of 1,008 distinct movie titles. The movie data set will be referred to as C1km in the following, and the movie names are available for download.^14^

### Acquiring tweets

To gather posts related to the two domains under assessment, i.e., music and movies, we use Twitter’s API^15^ to issue queries according to the schemes indicated in Table 2. Accounting for the time-varying behavior of the search results and to obtain a broad coverage, we queried Twitter from December 2010 to February 2011 and aggregated the posts retrieved over time for each query. The resulting set of tweets per query/named entity is then pre-processed by employing casefolding and stopping. When using the term sets TS_A and TS_S, stemming is employed additionally.

For artist set C224a, we achieved a coverage of 100%; for set C3ka, we achieved a coverage of 96.87%, i.e., for 2,906 artists out of the 3,000 tweets were available. Coverage for the movie data set C1km was considerably lower (82.8% or 834 movies), likely due to the fact that IMDb always lists the full, official movie title, which is often replaced by a shortened version when referring to the movie in a microblog, e.g., “The Fog of War: Eleven Lessons from the Life of Robert S. McNamara”.

As for the total amounts of tweets extracted, using collection C224a, 21,336 tweets were gathered for QS_A and 10,867 for QS_M. For set C3ka, 3,161,582 tweets were retrieved for QS_A and 2,972,130 for QS_M. For the movie set C1km, we retrieved 11,684,074 tweets using query scheme QS_A and 4,958,223 tweets using query scheme QS_M.

### Experimental setup

To assess the quality of the named entity’s term models, we perform *genre classification* experiments, evaluating the different algorithmic choices. As ground truth the genre labels given by allmusic.com and IMDb are used for the music sets and the movie set, respectively. Although genre taxonomies are often inconsistent and erroneous (Pachet and Cazaly 2000), it has become commonplace to use genre as a proxy for similarity. In principle, a more precise ground truth could be established from human similarity judgments. Complete similarity judgments are, however, not publicly available on a large scale, neither for music, nor for movies. Hence, we have to restrict evaluation to the retrieval task of determining *k* artists/movies similar to a given query artist/movie. This task resembles *k* nearest neighbor (kNN) classification, where the class of a seed item is predicted as the most frequent class among the seed’s *k* most similar items. In the case of the single-class classification problem given by the music data sets, performing kNN is straightforward. However, when dealing with multiple labels/classes assigned to each item, as in the case of the movie set, we opted to employ a strict decision rule: Given a seed item with *s* class labels associated and a number of *k* nearest neighbors to consider, we accumulate the number of occurrences of up to *s* classes among the *k* neighbors. We then calculate the (proportionate) precision of the top *s* classes given by the accumulated counts on the seed’s *s* classes, i.e., each of the top *s* classes among the *k* nearest neighbors that match one of the seed’s *s* classes account for a precision score of 1/*s*. The algorithm used to compute precision@*k* for the multi-class experiments is illustrated in Algorithm 1.

**Algorithm 1:**
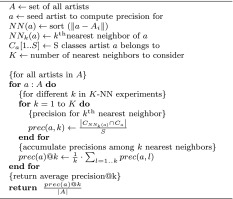
Precision@*k* in the multi-class case

We performed a two-staged evaluation: In order to determine and filter inferior algorithmic combinations, we first ran a comprehensive set of evaluation experiments on the equally genre-distributed data set C224a. In a second set of experiments, we then evaluated the remaining variants on the real-world artist set C3ka. On the movie set C1km all variants were evaluated.

Our experimental setting resembles the ones employed in Buckley and Voorhees (2000), Sanderson and Zobel (2005). Given a query item, the retrieval task is to find items of the same class(es) via similarity. We use *Mean Average Precision* (MAP) as performance measure. Employing Algorithm 1, MAP is simply computed as the arithmetic mean of the precision@*k* scores. Following Sanderson and Zobel (2005), we first calculate MAP of each distinct algorithmic setting on data set C224a. Excluding redundant combinations, a total of 23,100 single experiments have been conducted for set C224a and 11,627 for set C1km. In the first stage of the experiments, only variants that fulfill at least one of the following two conditions are retained:

- there is a relative MAP difference of 10% or less to the top-ranked variant
- or the *t* test does not show a significant difference to the top-ranked variant (at 5% significance level).

**Fig. 1:**
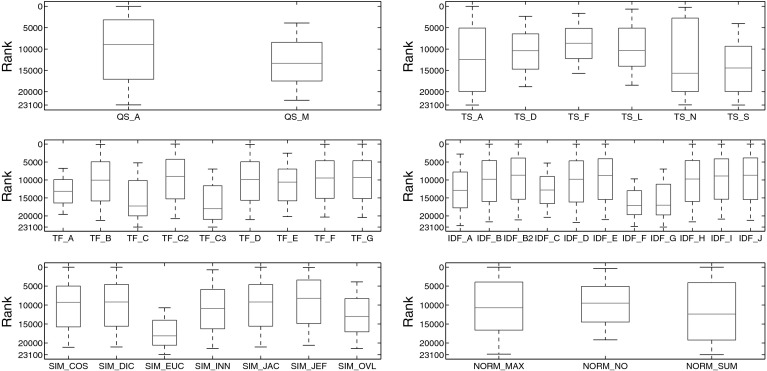
Box plots of ranks for each algorithmic choice on music set C224a

**Fig. 2:**
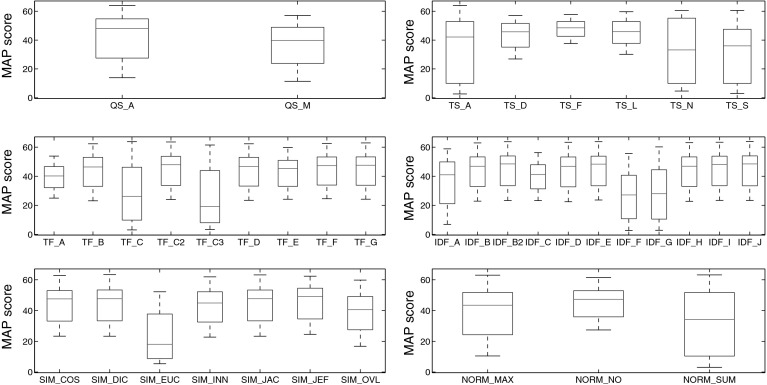
Box plots of MAP scores for each algorithmic choice on music set C224a

For set C224a, the top 577 variants have a relative MAP difference (from the 1st to the respective rank, taking the respective rank as basis) of less than 10%. The pairwise *t* test shows a significant difference for the top-ranked 1,809 variants. For the second stage of experimentation, conducted on collection C3ka, we therefore evaluated only these top-ranked 1,809 variants. For the movie set C1km, these numbers are 2,392 (relative MAP difference) and 5,629 (*t* test), respectively (Figs. 1, 2).

### Results and discussion

#### MAP scores

**Fig. 3:**
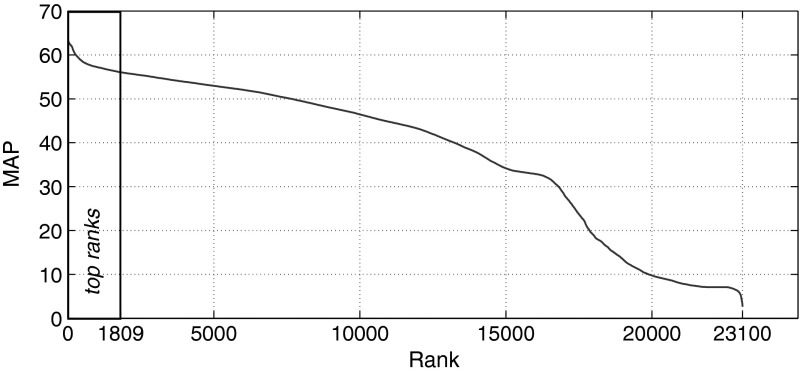
Distribution of MAP scores among all 23,100 ranks on music set C224a

Table 10 shows the 10 top-ranked and the 10 bottom-ranked variants with their MAP scores (considering up to 15 nearest neighbors) for set C224a. The MAP scores of the 23,100 evaluated variants span a wide range and are quite diverse (cf. Fig. 3), with a mean of μ = 37.89 and a standard deviation of σ = 17.16. From Table 10 it can be seen that highest MAP scores can only be achieved when using QS_A, TS_A, and NORM_NO. At the other end of the ranking we see that QS_M and SIM_OVL dominate the most inferior variants.

**Table 10:** MAP scores of the top-ranked and bottom-ranked variants on music set C224a

MAP	Variant
64.018	QS_A.TS_A.NORM_NO.TF_C2.IDF_E.SIM_JAC
63.929	QS_A.TS_A.NORM_NO.TF_C2.IDF_J.SIM_JAC
63.839	QS_A.TS_A.NORM_NO.TF_C.IDF_E.SIM_JAC
63.810	QS_A.TS_A.NORM_NO.TF_C2.IDF_E.SIM_COS
63.780	QS_A.TS_A.NORM_NO.TF_C.IDF_E.SIM_COS
63.780	QS_A.TS_A.NORM_NO.TF_C2.IDF_B2.SIM_JAC
63.780	QS_A.TS_A.NORM_NO.TF_C2.IDF_B2.SIM_DIC
63.720	QS_A.TS_A.NORM_NO.TF_C2.IDF_E.SIM_DIC
63.601	QS_A.TS_A.NORM_NO.TF_C2.IDF_J.SIM_COS
63.542	QS_A.TS_A.NORM_NO.TF_C.IDF_J.SIM_JAC
$\cdots$	$\cdots$
3.482	QS_M.TS_A.NORM_MAX.TF_G.IDF_G.SIM_OVL
3.452	QS_M.TS_S.NORM_SUM.TF_B.IDF_F.SIM_OVL
3.423	QS_M.TS_A.NORM_SUM.TF_C3.IDF_J.SIM_OVL
3.363	QS_M.TS_S.NORM_MAX.TF_G.IDF_F.SIM_OVL
3.274	QS_M.TS_A.NORM_SUM.TF_C.IDF_E.SIM_OVL
3.065	QS_M.TS_A.NORM_SUM.TF_C.IDF_J.SIM_OVL
3.006	QS_M.TS_A.NORM_MAX.TF_G.IDF_F.SIM_OVL
2.976	QS_M.TS_S.NORM_MAX.TF_F.IDF_F.SIM_OVL
2.857	QS_M.TS_A.NORM_MAX.TF_F.IDF_G.SIM_OVL
2.649	QS_M.TS_A.NORM_MAX.TF_F.IDF_F.SIM_OVL

Table 11 shows the top- and bottom-ranked variants with their MAP scores for the movie data set C1km (considering up to 50 nearest neighbors). Note that these MAP scores are overall lower than the scores for the music collections, with a mean of μ = 23.12 and a standard deviation of σ = 2.61. This lower overall performance is partly due to the higher number of classes, partly because of the stricter decision rule employed in the classification process, cf. Sect. 4.3. Highest ranks are again dominated by query scheme QS_A and term set TS_A, whereas the lowest-ranking variants are dominated by QS_A.TS_S.NORM_SUM.SIM_JEF.

**Table 11:** MAP scores of the top-ranked and bottom-ranked variants on movie set C1km

MAP	Variant
27.964	QS_A.TS_A.NORM_SUM.TF_G.IDF_C.SIM_INN
27.962	QS_A.TS_A.NORM_NO.TF_A.IDF_C.SIM_DIC
27.962	QS_A.TS_A.NORM_NO.TF_A.IDF_C.SIM_JAC
27.962	QS_A.TS_A.NORM_MAX.TF_A.IDF_C.SIM_DIC
27.962	QS_A.TS_A.NORM_MAX.TF_A.IDF_C.SIM_JAC
27.895	QS_A.TS_A.NORM_NO.TF_E.IDF_C.SIM_DIC
27.895	QS_A.TS_A.NORM_NO.TF_E.IDF_C.SIM_JAC
27.895	QS_A.TS_A.NORM_SUM.TF_E.IDF_C.SIM_DIC
27.895	QS_A.TS_A.NORM_SUM.TF_E.IDF_C.SIM_JAC
27.895	QS_A.TS_A.NORM_MAX.TF_E.IDF_C.SIM_DIC
$\cdots$	$\cdots$
17.101	QS_M.TS_S.NORM_SUM.TF_C3.IDF_H.SIM_JEF
17.101	QS_M.TS_S.NORM_SUM.TF_C3.IDF_I.SIM_JEF
17.101	QS_M.TS_S.NORM_SUM.TF_D.IDF_F.SIM_JEF
17.101	QS_M.TS_S.NORM_SUM.TF_D.IDF_G.SIM_JEF
17.101	QS_M.TS_S.NORM_SUM.TF_E.IDF_F.SIM_JEF
17.101	QS_M.TS_S.NORM_SUM.TF_E.IDF_G.SIM_JEF
17.101	QS_M.TS_S.NORM_SUM.TF_F.IDF_F.SIM_JEF
17.101	QS_M.TS_S.NORM_SUM.TF_F.IDF_G.SIM_JEF
17.101	QS_M.TS_S.NORM_SUM.TF_G.IDF_F.SIM_JEF
17.101	QS_M.TS_S.NORM_SUM.TF_G.IDF_G.SIM_JEF

When comparing Tables 10 and 11, it becomes obvious that the best- and worst-performing variants vary considerably with the set of names entities, in particular in terms of *TF* and *IDF* formulations as well as similarity measures. Furthermore, it seems easier to identify algorithmic choices that yield worse performance and should thus be avoided than to clearly suggest best-performing choices.

#### Distribution of specific algorithmic choices

**Fig. 4:**
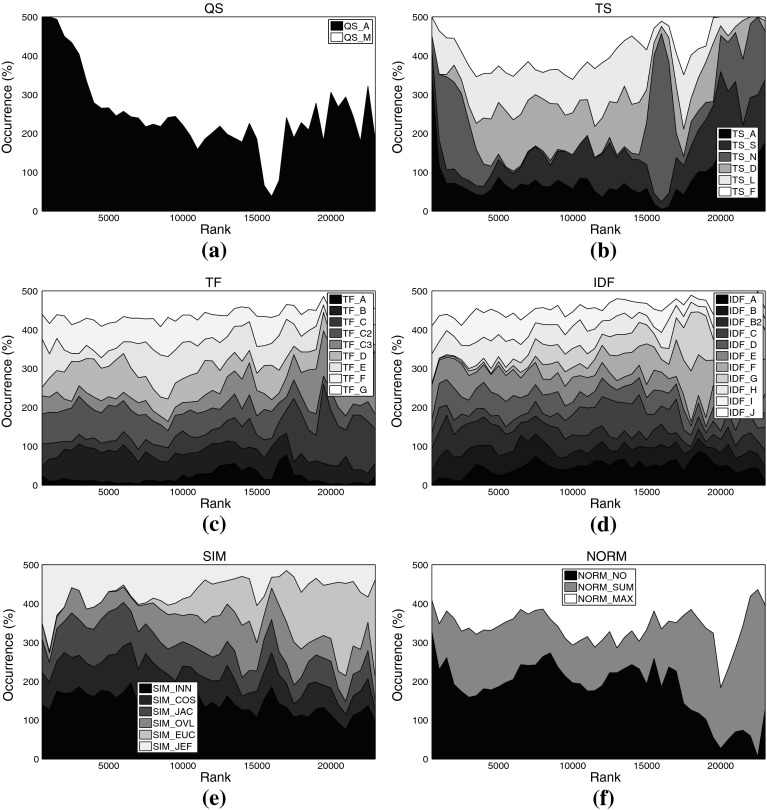
Distribution of different settings among *all variants* on music set C224a. **a** Query scheme, **b** term set, **c** *TF* formulation, **d** *IDF* formulation, **e** similarity function, **f** normalization method

**Fig. 5:**
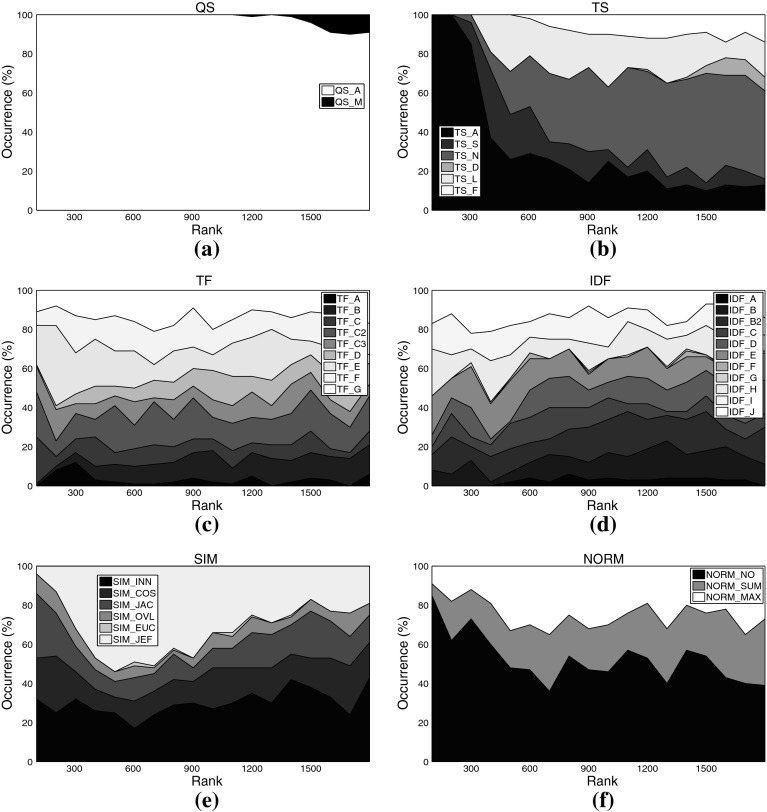
Distribution of different settings among the *top-ranked variants* on music set C224a. **a** Query scheme, **b** term set, **c** *TF* formulation, **d** *IDF* formulation, **e** similarity function, **f** normalization method

**Fig. 6:**
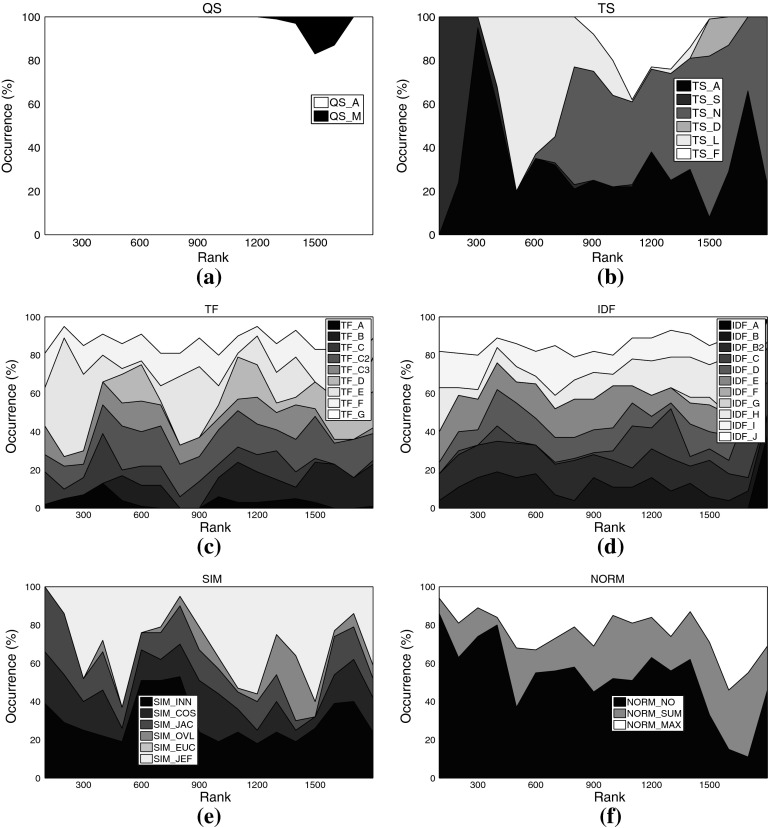
Distribution of different settings among the *top-ranked variants* on music set C3ka. **a** Query scheme, **b** term set, **c** *TF* formulation, **d** *IDF* formulation, **e** similarity function, **f** normalization method

**Fig. 7:**
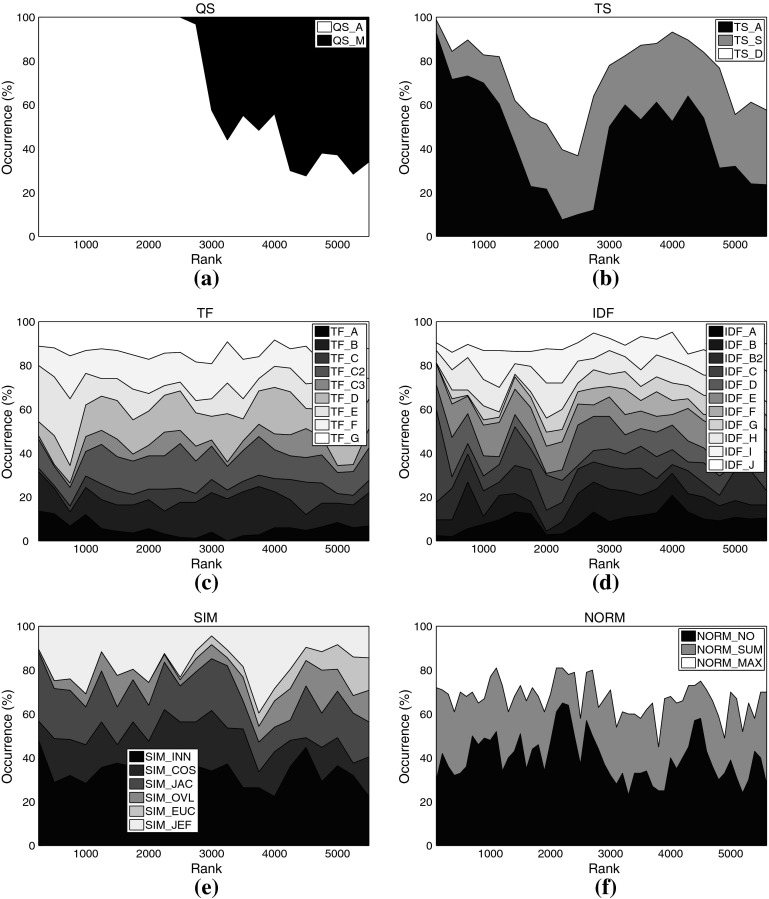
Distribution of different settings among the *top-ranked variants* on movie set C1km. **a** Query scheme, **b** term set, **c** *TF* formulation, **d** *IDF* formulation, **e** similarity function, **f** normalization method

Figure 4 displays the distribution of each analyzed aspect among all 23,100 experimental setups investigated for set C224a. Figure 5 shows this distribution among the 1,809 top-ranked variants. Figure 6 shows the top-ranked algorithmic choices for artist set C3ka and Fig. 7, eventually, shows this distribution for the movie data set C1km.

For some aspects, general rules can be derived from these plots: Regarding the query scheme, it is obvious that using only the named entity as indicator to determine related tweets (QS_A) outperforms adding domain-specific key words. This result at first glance contrasts earlier work on Web-based music artist classification (Knees et al. 2008). However, Knees et al. analyze Web pages, not microblogs. It seems that adding any additional key word too strongly prunes Twitter’s result set.

As for the term sets used for indexing, the very top ranks are dominated by algorithmic variants that use the complete set of terms occurring in the corpus (TS_A), for both the music and the movie data sets. It is noteworthy, however, that the good performance of the general term sets (TS_A and TS_S) comes at the price of much higher computational complexity (cf. Tables 3, 4 for term set cardinalities). Hence, when performance is crucial, the results suggest using other term sets. A particularly good choice when the domain is music at first glance seems to be TS_N, the list of artist names, as it is the set that most frequently occurs among the top-ranked variants (32.5% or 588 times). However, TS_N yields very unstable results, as will be shown in the subsequent subsection. Another interesting finding is that the music dictionary TS_D, despite its good performance for similarity-based artist clustering using *Web pages*, cf. Pampalk et al. (2005), occurs first only at rank 1,112. An empirically verified reason for this may be that Twitter users tend to refrain from using a decent music-specific vocabulary, even when they twit about music-related issues.^16^ For the movie set C1km, in contrast, TS_D represents a good trade-off between computational complexity and accuracy as it does not significantly more seldom occur among the top-ranked variants than the set TS_S (both about 28 vs. 44% for TS_A). It seems that a collaboratively assembled dictionary, such as TS_D for the movie domain, outperforms a domain-specific one assembled by experts, such as TS_D for the music domain, provided it is not too small.

As for the term weighting functions (*TF* and *IDF* variants), no clear picture regarding favorable variants emerges when analyzing the top-ranked algorithmic combinations. We found, however, that TF_A occurred most seldom among the top-ranked variants, regardless of the data set. This variant should thus be avoided. The most frequently occurring formulations on the other hand are TF_C2 (15.69% of the top-ranks for the music sets) and TF_E (16.80%), the latter being particularly present in the very top ranks for the music data sets. TF_C2 also occurs frequently among the top-ranked variants of the movie set C1km (13.52%), together with TF_D (14.55%), TF_F (13.82%), and TF_G (13.87%).

Analogously to *TF*, for *IDF* variants we can easily point to formulations that should be avoided, namely IDF_G (0.50% among C3ka’s top ranks), IDF_F (0.66%), and IDF_A (2.54%). However, we were not able to determine a single variant that clearly outperforms all others. The *IDF* variants most frequently occurring within the top ranks of the music sets are IDF_B2 (13.93%), IDF_J (13.71%), and IDF_E (13.38%). For the movie set C1km, the very same variants perform best (IDF_E with 11.16% occurrence, IDF_J with 11.09%, and IDF_B2 with 9.95%).

As for the similarity measure, we found no clear evidence that cosine similarity (SIM_COS), the de-facto standard measure in IR, generally outperforms the others. It is likely that the key advantage of SIM_COS, the document length normalization, plays a minor role here, because tweets are limited to 140 characters which are usually exhausted by Twitter users. Further support for this hypothesis is given by the remarkably good performance of the simple inner product SIM_INN measure that does not perform any length normalization. On all three data sets, SIM_INN occurs almost twice as often as SIM_COS among the top-ranked variants (about 32 vs. 16%). Also among the virtual document normalization methods, using no normalization at all (NORM_NO) outperforms the other variants investigated, accounting for 52.24% of the top ranks for the music sets, and for 39.94% of the top variants using set C1km. In addition to SIM_INN, also the Jeffrey divergence-based similarity SIM_JEF performed comparably well over all data sets (31.5% for the music sets, 17.77% for C1km).

To investigate if extrapolating the results from the small music set C224a to the real-world set C3ka is valid, we calculated Spearman’s rank-order correlation coefficient (e.g., Sheskin 2004) on the two rankings obtained with the two artist sets. The computation revealed a moderate correlation of 0.37. This correlation indicates that the rankings produced by the same algorithmic choices are not largely influenced by factors such as size of artist collection or number of artists per genre.

#### Average quality and performance variance

In order to assess the quality of individual algorithmic choices—e.g., the use of a specific similarity measure—for the overall task of retrieving similar items, we further computed for all aspects analyzed and for each concrete choice average performance measures over all combinations that use the algorithmic choice under consideration. In particular, *arithmetic mean*, *median*, and *standard deviation* of the *ranks* and the actual *MAP scores* were calculated; mean and median describe the overall performance of each algorithmic choice, whereas the standard deviation can be interpreted as an estimate of the “robustness” of the algorithmic choice against changes in other algorithmic aspects. If variants employing a specific choice are tightly grouped together in the rank-ordered set of all combinations, their standard deviation will be small. This also means that the performance of such tightly grouped variants (according to a particular aspect, e.g., use of term set TS_F) is less sensitive to changes in other choices (for example, employing a different normalization).

To investigate both average performance and robustness of specific variants, Fig. 1 shows box plots of the rankings obtained for each algorithmic choice in each of the six broad aspects under consideration.^17^ Figure 2 shows the same statistical figures, but this time computed on MAP scores instead of ranks. Table 12 reports detailed results for each algorithmic choice.

**Table 12:** Detailed results among algorithmic choices on music set C224a

Variant	Rank					MAP				
	Median	Mean	SD	Min	Max	Median	Mean	SD	Min	Max
QS_A	**8,929**	**10,021**	7,247.2	**1**	**23,080**	**48.066**	**40.380**	18.016	**3.869**	**64.018**
QS_M	13,340	13,080	**5,633.8**	1,112	23,100	39.732	35.399	**15.866**	2.649	57.083
TS_A	12,421	12,257	7,869.8	**1**	23,100	42.158	34.325	20.659	2.649	64.018
TS_D	10,388	10,983	5,349.6	1,112	23,052	45.774	40.693	14.028	4.851	57.083
TS_F	**8,639**	**9,068**	**4,831.1**	529	**19,098**	**48.527**	**45.408**	**10.402**	**12.857**	**58.393**
TS_L	10,343	9,949	5,561.6	318	23050	45.863	43.012	12.610	4.970	59.702
TS_N	15,660	12,880	7,901.3	221	23,058	33.214	33.181	18.853	4.702	60.595
TS_S	14,444	14,165	6,439.0	225	23,098	35.997	30.717	18.527	2.976	60.536
TF_A	13,152	12,310	**5,325.9**	144	**22,979**	40.223	38.829	**12.361**	**5.982**	61.875
TF_B	10,034	10,505	6,373.3	90	23,092	46.399	40.625	15.846	3.452	62.321
TF_C	17,240	14912	6,462.9	3	23,096	26.116	28.360	18.222	3.066	63.839
TF_C2	**9,006**	**9,854**	6,509.2	**1**	23,088	**47.961**	**41.976**	15.709	3.631	**64.018**
TF_C3	17,972	15,421	6,618.3	22	23,093	19.137	26.607	18.628	3.423	63.066
TF_D	9,871	10,371	6,290.5	89	23,053	46.682	40.974	15.697	4.821	62.321
TF_E	10,587	10,981	6,110.3	15	23,063	45.417	40.524	14.978	4.435	63.184
TF_F	9,448	10,079	6,276.5	62	23,100	47.321	41.836	15.187	2.649	62.589
TF_G	9,274	10,028	6,303.5	25	23,097	47.589	41.899	15.288	3.006	62.976
IDF_A	12,855	12,632	5,775.5	449	23,055	41.012	36.061	15.921	4.792	58.839
IDF_B	9,773	10,475	6,681.4	29	23,000	46.860	40.374	16.573	5.804	62.946
IDF_B2	**8,665**	9,780	6,765.0	6	23,058	**48.482**	41.785	16.607	4.702	63.780
IDF_C	12,782	12,409	6,020.9	87	23,027	41.220	37.195	15.609	5.446	62.351
IDF_D	9,790	10,554	6,700.2	13	23,015	46.845	40.208	16.601	5.595	63.363
IDF_E	8,744	9,784	6,750.8	**1**	23,095	48.363	41.722	16.674	3.274	**64.018**
IDF_F	17,108	16,061	**4,884.2**	459	23,100	27.173	26.772	15.586	2.649	58.750
IDF_G	17,009	15,439	5,334.3	258	23,099	28.021	28.219	**16.568**	2.857	60.179
IDF_H	9,731	10,304	6,596.3	19	**22,970**	46.920	40.977	16.472	**6.042**	63.155
IDF_I	8,889	9,887	6,637.9	12	23,038	48.125	41.664	16.435	5.298	63.393
IDF_J	8,673	**9,731**	6,750.9	2	23,096	**48.482**	**41.807**	16.660	3.066	63.929
SIM_COS	9,281	10,275	6,463.8	4	23,060	47.559	41.047	16.073	4.583	63.809
SIM_DIC	9,201	10,127	6,471.4	7	**22,910**	47.679	41.323	16.013	**6.339**	63.780
SIM_EUC	18,116	17,262	**4,082.2**	562	23,027	18.095	23.070	**14.796**	5.446	58.274
SIM_INN	10,896	11,135	6,407.8	141	23,005	44.926	39.429	16.049	5.774	61.905
SIM_JAC	9,194	10,132	6,470.0	**1**	22,972	47.708	41.318	16.017	6.042	**64.018**
SIM_JEF	**8,235**	**9,299**	6,820.6	87	23,058	**49.137**	**42.635**	16.488	4.702	62.351
SIM_OVL	13,004	12,624	6,074.8	18	23,100	40.610	36.403	16.155	2.649	63.155
NORM_MAX	11,851	11,781	6,415.5	27	23,100	43.452	37.418	16.919	2.649	62.976
NORM_NO	**9,482**	**9,811**	**5,908.0**	**1**	**23,054**	**47.292**	**43.276**	**13.289**	**4.821**	**64.018**
NORM_SUM	14,978	13,277	7,219.1	15	23,096	34.241	32.300	19.258	3.066	63.184

Best results for each category are printed in boldface

Taking a closer look at Figs. 1, 2 and Table 12, the following observations can be made:

- QS_A clearly outperforms QS_M in terms of quality, although the results obtained with QS_M are more robust.
- TS_F outperforms all other term sets, both in quality and robustness. This superiority becomes even more clearly visible when using MAP scores as quality measure (Fig. 2) instead of ranks (Fig. 1). Interestingly, term sets TS_A and TS_N do not perform well overall, since the results they produce are spread across a wide range of ranks (or MAP values), and their quality is not too good either. Figure 4b reveals the reason for the huge spread of TS_N: Even though TS_N is employed in some of the highest ranked variants, there are also two large clusters of variants employing TS_N towards the very end of the rank-ordered set of experiments (> rank 15,000). Especially its combination with the algorithmic choices QS_M, TF_B, TF_D, TF_E, IDF_A, IDF_H, SIM_INN, or SIM_OVL proves detrimental. Looking at the quality scores of TS_A, a particularly interesting fact stands out, which is that TS_A performs much better in terms of MAP than in terms of rank score. Hence, although the findings presented in Sect. 4.4.2 suggest that TS_A is well-suited to yield top results, this seems to be true only when particular other algorithmic choices are present. As a consequence, TS_A should be used with caution, only when computational complexity is not an issue and when other algorithmic choices can be ensured (cf. Table 10).
- As for the term frequency, formulations TF_C and TF_C3 perform poorly and are unstable. We therefore strongly recommend to refrain from these. The binary formulation TF_A is the most stable one, but performs inferior to all but the worst variants mentioned above. Among the other, preferably performing variants, TF_C2 sticks out as yielding particularly good results, in terms of both rank score and MAP. Furthermore, TF_F and TF_G perform equally well as TF_C2 in terms of MAP and slightly worse than the top-performing variant in terms of rank score. Both TF_F and TF_G are slightly more robust than TF_C2. Hence, as an overall recommendation one should select one of the term frequency formulations TF_C2, TF_F, or TF_G, with a slight preference for the former one if top-performance is crucial and a slight preference for one of the latter two variants if stability of the results is more important.
- Variants IDF_A, IDF_C, IDF_F, and IDF_G perform significantly worse than the other formulations of inverse document frequency. As for top-performing choices, IDF_E ranks at the very top according to both MAP and rank scores. Also IDF_B2 and IDF_J are not significantly inferior.
- Among the similarity functions, SIM_EUC performs remarkably inferior to all other variants. SIM_OVL does not perform considerably better. Best results can be achieved employing SIM_JEF, while at the same time maintaining a reasonable stability level.
- NORM_NO performs best in terms of quality and robustness, whereas NORM_SUM performs worst in both regards.

#### Comparison with web page-based experiments

We also conducted a similar study using as data source Web pages related to music artists instead of microblogs (Schedl et al. 2011). In order to assess the specificities of microblogs, in the following the results obtained in the paper at hand for the music data sets are compared against those reported in Schedl et al. (2011), where the same evaluation setting is employed. Although the music data sets are partly different, the results of Schedl et al. (2011) are comparable to those of the current study. Overall, the best-performing variants according to Schedl et al. (2011) in this paper’s notation are the following:

- TF_C3.IDF_I.SIM_COS
- TF_C3.IDF_H.SIM_COS
- TF_C2.IDF_I.SIM_COS
- TF_C2.IDF_H.SIM_COS

In all top-ranked variants, no normalization on the Web page-level, i.e., giving each Web page retrieved for the artist under consideration the same weight, is performed. Nevertheless, the virtual documents are normalized, i.e., when aggregating individual Web pages retrieved for a particular artist to a virtual document, each term score is divided by the absolute number of Web pages retrieved for the artist that contain the term.

Comparing the two studies, the first observation to be made is that regardless of the data source (Web pages or microblogs), logarithmic formulations of *TF* tend to perform best (in particular for music artists). As for *IDF*, the variants IDF_I, IDF_H, and IDF_B2 perform best for Web pages, while IDF_B2, IDF_E, and IDF_J yield highest MAP scores for microblogs. Thus, again logarithmic formulations considerably outperform other variants for both data sources. Regarding the similarity measure, the top-ranked variants on the corpus of Web pages employ cosine similarity, while for microblogs no clear indication for the cosine measure to outperform the others can be found. Furthermore, normalization does not improve results when the corpus is constituted of tweets. In contrast, when the corpus comprises Web pages, normalization on the level of virtual documents considerably ameliorates the MAP scores. No comparison can be made on the level of term sets due to the fact that Schedl et al. (2011) does not take into account different dictionaries for indexing. As for the query scheme, QS_M which includes the term “music” in addition to the named entity sought for clearly outperforms QS_A on the Web-page-corpus, while the inverse holds on the microblog-corpus. It seems that adding additional, domain-specific search terms to the query is counterproductive when looking for microblogs since it prunes the set of tweets too heavily, while doing so is a necessity to filter unrelated Web pages from the search results.

### Alternative classifiers

Since we modeled and evaluated the retrieval task as a genre classification task, we can alternatively use classifiers other than kNN for evaluation purposes. We hence compare the memory-based kNN classifier with several state-of-the-art classifiers: the kernel-based *Support Vector Machines* (SVM) (Vapnik 1995), *Random Forests* (RF) (Breiman 2001), i.e., an ensemble learner based on decision trees, and *Repeated Incremental Pruning to Produce Error Reduction* (RIPPER) (Cohen 1995), a propositional rule learner. We employed 10-fold Cross-Validation using the default parameters of the respective WEKA (Hall et al. 2009) classifiers.

Tables 13, 14, and 15 show the highest- and lowest-ranked variants when using as classifier SVM, RF, and RIPPER, respectively. Similar to the kNN experiments described in Sect. 4.3, query set QS_A clearly outperforms QS_M. It can be observed that SVM benefits from having access to as much data as possible, i.e., it achieves highest accuracies when operating on term set TS_A. The Random Forest classifier yields significantly lower accuracies and performs best when using artist names as term set TS_N. The rule learner RIPPER seemingly performs best on the Freebase set TS_F, the reason for which is probably the clearest semantic distinction between the terms in this dictionary. Performing no normalization proved beneficial also for classifiers other than kNN, although in the case of RF, this becomes apparent better from looking at the top ranks in Fig. 9e than from Table 14. To yield top performance with the RF classifier, the use of IDF_F (in addition to QS_A.TS_N) seems to be more important than employing a particular normalization function. No clear picture emerges, in contrast, when analyzing the impact of the term frequency formulation. Even though the top 4 performers with SVM employ variants of the TF_C formulation, combinations including several other formulations can be found among the top-performing variants as well. Among the top-performing variants in the RF experiments, the simple binary match function TF_A appears surprisingly often. For the decision tree learner, the *IDF* formulation hence seems to be more important. For RIPPER, variants of TF_C clearly outperform all other choices.

**Table 13:** Accuracies of the top-ranked and bottom-ranked variants using SVM classification on music set C224a

Acc.	Variant
71.875	QS_A.TS_A.NORM_NO.TF_C.IDF_G
71.875	QS_A.TS_A.NORM_NO.TF_C2.IDF_G
71.429	QS_A.TS_A.NORM_NO.TF_C3.IDF_G
70.089	QS_A.TS_A.NORM_NO.TF_C2.IDF_F
69.643	QS_A.TS_A.NORM_MAX.TF_F.IDF_G
69.196	QS_A.TS_A.NORM_NO.TF_C.IDF_F
68.750	QS_A.TS_A.NORM_MAX.TF_B.IDF_F
68.750	QS_A.TS_A.NORM_MAX.TF_D.IDF_F
68.750	S_A.TS_A.NORM_MAX.TF_G.IDF_F
68.750	QS_A.TS_A.NORM_MAX.TF_G.IDF_G
68.750	QS_A.TS_A.NORM_NO.TF_D.IDF_F
68.750	QS_A.TS_A.NORM_SUM.TF_D.IDF_F
$\cdots$	$\cdots$
7.143	QS_A.TS_S.NORM_MAX.TF_A.IDF_F
7.143	QS_A.TS_S.NORM_MAX.TF_E.IDF_F
7.143	QS_A.TS_S.NORM_NO.TF_A.IDF_F
7.143	QS_A.TS_S.NORM_NO.TF_E.IDF_F
7.143	QS_A.TS_S.NORM_NO.TF_F.IDF_F
7.143	QS_A.TS_S.NORM_NO.TF_G.IDF_F
7.143	QS_A.TS_S.NORM_SUM.TF_A.IDF_F
7.143	QS_A.TS_S.NORM_SUM.TF_E.IDF_F
6.696	QS_A.TS_S.NORM_MAX.TF_C.IDF_F
6.696	QS_A.TS_S.NORM_MAX.TF_C3.IDF_F

**Table 14:** Accuracies of the top-ranked and bottom-ranked variants using RF classification on music set C224a

Acc.	Variant
57.589	QS_A.TS_N.NORM_MAX.TF_A.IDF_F
57.589	QS_A.TS_N.NORM_SUM.TF_E.IDF_F
57.589	QS_A.TS_N.NORM_NO.TF_A.IDF_F
57.589	QS_A.TS_N.NORM_SUM.TF_A.IDF_F
57.589	QS_A.TS_N.NORM_MAX.TF_E.IDF_F
57.589	QS_A.TS_N.NORM_NO.TF_E.IDF_F
57.143	QS_A.TS_N.NORM_SUM.TF_G.IDF_F
56.696	QS_A.TS_N.NORM_NO.TF_F.IDF_F
55.357	QS_A.TS_N.NORM_MAX.TF_C2.IDF_F
54.911	QS_A.TS_N.NORM_NO.TF_A.IDF_G
54.911	QS_A.TS_N.NORM_NO.TF_A.IDF_G
54.911	QS_A.TS_N.NORM_SUM.TF_A.IDF_G
54.911	QS_A.TS_N.NORM_MAX.TF_A.IDF_G
54.911	QS_A.TS_N.NORM_MAX.TF_C2.IDF_G
54.911	QS_A.TS_N.NORM_SUM.TF_C2.IDF_G
$\cdots$	$\cdots$
7.589	QS_A.TS_S.NORM_MAX.TF_C2.IDF_F
7.143	QS_A.TS_S.NORM_SUM.TF_A.IDF_F
7.143	QS_A.TS_S.NORM_MAX.TF_A.IDF_F
7.143	QS_A.TS_S.NORM_MAX.TF_F.IDF_F
7.143	QS_A.TS_S.NORM_NO.TF_B.IDF_F
7.143	QS_A.TS_S.NORM_SUM.TF_C2.IDF_F
7.143	QS_A.TS_S.NORM_NO.TF_A.IDF_F
7.143	QS_A.TS_S.NORM_NO.TF_C2.IDF_F
6.697	QS_A.TS_S.NORM_SUM.TF_G.IDF_F
6.697	QS_A.TS_S.NORM_SUM.TF_B.IDF_F
6.250	QS_A.TS_S.NORM_MAX.TF_C.IDF_F

**Table 15:** Accuracies of the top-ranked and bottom-ranked variants using RIPPER classification on music set C224a

Acc.	Variant
58.4821	QS_A.TS_F.NORM_NO.TF_B.IDF_H
58.4821	QS_A.TS_F.NORM_NO.TF_C3.IDF_H
58.0357	QS_A.TS_F.NORM_MAX.TF_C.IDF_C
58.0357	QS_A.TS_F.NORM_MAX.TF_C.IDF_D
58.0357	QS_A.TS_F.NORM_MAX.TF_C.IDF_E
58.0357	QS_A.TS_F.NORM_MAX.TF_C.IDF_J
57.5893	QS_A.TS_F.NORM_NO.TF_C.IDF_J
57.5893	QS_A.TS_F.NORM_NO.TF_C2.IDF_I
57.5893	QS_A.TS_F.NORM_NO.TF_C.IDF_A
57.5893	QS_A.TS_F.NORM_NO.TF_C2.IDF_A
57.5893	QS_A.TS_F.NORM_NO.TF_C.IDF_B2
57.5893	QS_A.TS_F.NORM_NO.TF_B.IDF_D
57.5893	QS_A.TS_F.NORM_NO.TF_C2.IDF_D
57.5893	QS_A.TS_F.NORM_NO.TF_B.IDF_C
57.5893	QS_A.TS_F.NORM_NO.TF_B.IDF_A
57.5893	QS_A.TS_F.NORM_NO.TF_C3.IDF_B
$\cdots$	$\cdots$
4.4643	QS_A.TS_S.NORM_SUM.TF_G.IDF_F
4.4643	QS_A.TS_S.NORM_NO.TF_E.IDF_F
4.4643	QS_A.TS_S.NORM_NO.TF_B.IDF_F
4.4643	QS_A.TS_S.NORM_SUM.TF_C.IDF_F
4.4643	QS_A.TS_D.NORM_MAX.TF_A.IDF_G
4.4643	QS_A.TS_S.NORM_NO.TF_G.IDF_F
4.4643	QS_A.TS_S.NORM_SUM.TF_B.IDF_F
4.4643	QS_A.TS_S.NORM_NO.TF_F.IDF_F
4.4643	QS_A.TS_S.NORM_SUM.TF_E.IDF_F
4.4643	QS_A.TS_S.NORM_SUM.TF_C2.IDF_F
4.4643	QS_A.TS_D.NORM_SUM.TF_A.IDF_G
4.4643	QS_A.TS_S.NORM_MAX.TF_G.IDF_F
4.4643	QS_A.TS_S.NORM_MAX.TF_C2.IDF_F
4.4643	QS_A.TS_S.NORM_NO.TF_A.IDF_F
4.4643	QS_A.TS_S.NORM_NO.TF_C2.IDF_F
4.4643	QS_A.TS_S.NORM_SUM.TF_D.IDF_F

**Fig. 8:**
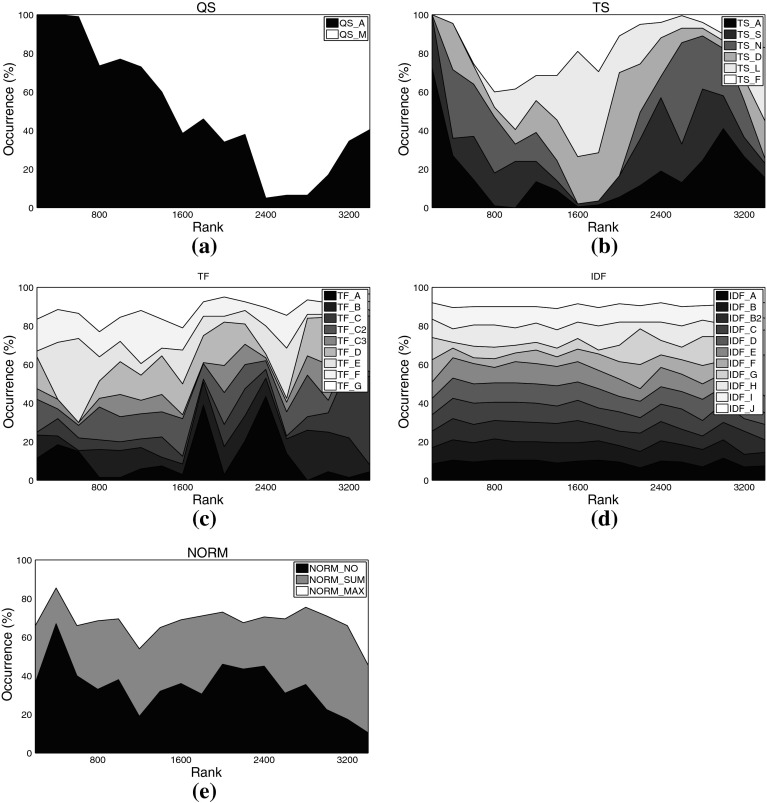
Distribution of different settings among *all variants* using SVM classification on music set C224a. **a** Query scheme, **b** term set, **c** *TF* formulation, **d** *IDF* formulation, **e** normalization method

**Fig. 9:**
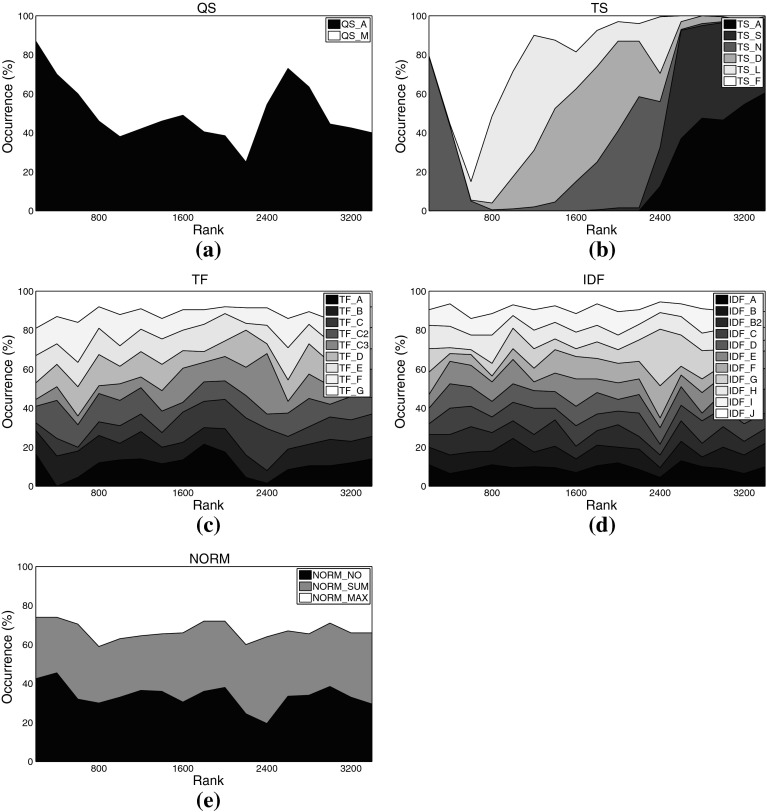
Distribution of different settings among *all variants* using RF classification on music set C224a. **a** Query scheme, **b** term set, **c** *TF* formulation, **d** *IDF* formulation, **e** normalization method

**Fig. 10:**
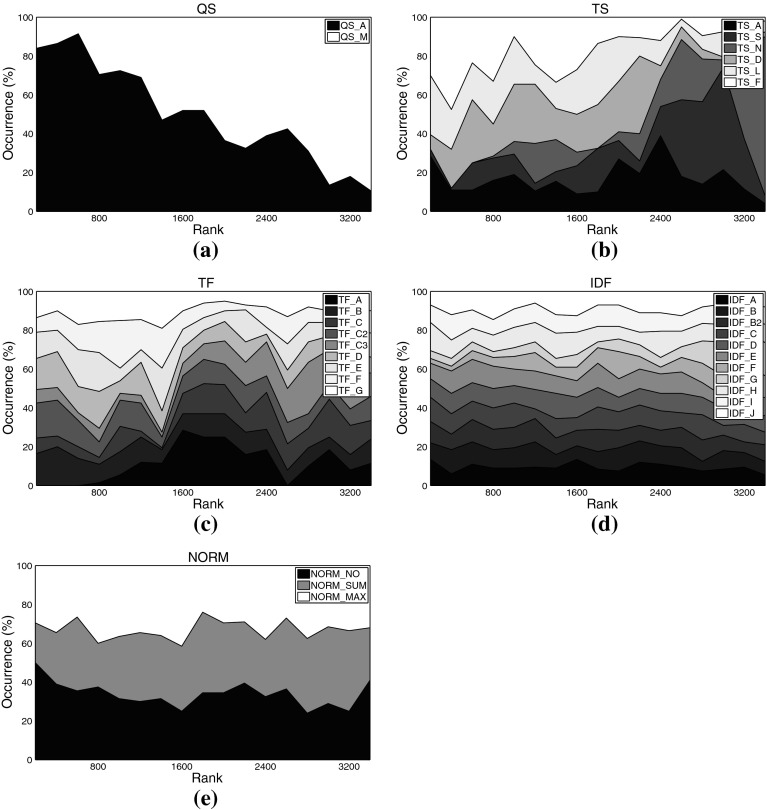
Distribution of different settings among *all variants* using RIPPER classification on music set C224a. **a** Query scheme, **b** term set, **c** *TF* formulation, **d** *IDF* formulation, **e** normalization method

Figures 8, 9, and 10 show the distribution of each algorithmic choice among all 3,564 experimental setups^18^ when using classifier SVM, RF, and RIPPER, respectively. Although these plots do not reveal significant information for all aspects analyzed, we can summarize the interesting observations and consequently formulate advices as follow:

- QS_A clearly outperforms QS_M with all classifiers.
- TS_A is found frequently among the top ranks in the SVM experiments, but also peaks at the very bottom ranks. The top 600 ranks of the RF experiments are entirely dominated by TS_N, and TS_F performs very well with the RIPPER classifier. The generic but broad vocabularies TS_A and TS_S perform remarkably inferior when using RF or RIPPER. It seems that rule learners and decision tree learners benefit from a smaller, but more well defined vocabulary, such as TS_N or TS_F.
- It is hard to give advice for favoring or refraining from specific choices of the term frequency function. When using an SVM classifier, Fig. 8c might suggest to employ TF_E or TF_G, because both are frequently found among the top-ranked variants; however, neither of them does consistently perform well. In particular TF_E also occupies inferior positions around rank 2,600. For the RF classifier, TF_G seems the most favorable *TF* formulation, too. One clear advice that can be given is to refrain from TF_A, regardless of the classifier applied. Even though binary match performs well in some settings, the peaks at mediocre and lowest ranks do by no means suggest the use of TF_A.
- The rather uniform distribution of the *IDF* variants among all ranks does not encourage the formulation of specific advices.
- Slight (when using RF or RIPPER) to rather dominant (SVM) peaks of the NORM_NO setting, correspond well to the observation already made for the kNN experiments using MAP as performance measure. Due to the special characteristics of tweets, it is not advisable to perform document length normalization.

To investigate whether results are consistent between different classifiers in terms of the variants’ rank-order according to classification accuracy, we computed Spearman’s rank-order correlation coefficient. The pairwise correlation can be found in Table 16. As it can be seen, different classifiers not very surprisingly yield different ranks for the same algorithmic variants. Nevertheless, a small but significant (*p* = 0.00002) correlation between SVM and RF could be observed. A moderate to high correlation between SVM and RIPPER is notable as well. Between RF and RIPPER a slight to moderate correlation is present. The *p* values for the combinations (SVM,RIPPER) and (RF,RIPPER) are infinitesimally small.

**Table 16:** Pairwise Spearman’s rank-order correlation coefficients between variants produced by alternative classifiers on music set C224a

	SVM	RF	RIPPER
SVM		0.071	0.528
RF	0.071		0.189
RIPPER	0.528	0.189	

## Conclusions and future work

In this article, we presented a comprehensive evaluation of using Twitter posts for the purpose of similarity estimation between named entities. To this end, we performed tens of thousands single experiments on three data sets, two related to the music domain, one from the movie domain. Different algorithmic choices related to query scheme, index term set, length normalization, $TF\cdot IDF$ formulation, and similarity measure were thoroughly investigated. The main findings can be summarized as follows:

- Restricting the search by domain-specific key words prunes the resulting set of tweets too heavily. Using only the named entity as query (QS_A) should be favored.
- Top-ranked results are achieved using all terms in the corpus (TS_A), though at high computational costs and little robustness against small changes in other algorithmic choices. If computational complexity or robustness is an issue, the results suggest using as index term set a domain-specific dictionary (TS_F for the music domain or TS_D for the movie domain).
- Normalizing for length does not significantly improve the results, neither when performed on term vectors, nor when included in the similarity function. Taking into account the higher computational costs, we therefore recommend refraining from normalization (NORM_NO) and using as similarity measure, for example, the inner product (SIM_INN) or the Jeffrey divergence-based similarity (SIM_JEF). Both SIM_EUC and SIM_OVL should definitively be avoided.
- The binary match *TF* formulation TF_A should not be used. The most favorable variants are TF_C2 and TF_E. But also TF_F and TF_G do not perform significantly worse, regardless of the data set used.
- Among the *IDF* formulations, we suggest to refrain from using IDF_A, IDF_F, and IDF_G, as they performed poorly on all data sets. Better alternatives are given by formulations IDF_B2, IDF_E, and IDF_J, which ranked well on all sets.

Future work on evaluating different similarity models based on microblogs will include incorporating the bloger’s perspective, for example, by exploiting social graphs. Taking into account that perceived similarities are often subjective, influenced by peers, and can be defined according to very different dimensions, in the music as well as in the movie domain, a more fine-grained analysis based on the results presented here should be performed. As some of the algorithmic choices of the best- and worst-performing combinations varied between the movie and music data sets, we further plan to assess if the performance of specific variants depends on the type of the named entities. We will therefore conduct experiments on other sets of named entities, for example, politicians or books.

Another promising research direction is assessing *temporal and geographic properties of tweets*. Geographical aspects could be used, for example, to develop *geo-aware popularity estimates* of named entities. Together with temporal information, such a popularity measure could give indication on the development and spreading of trends around the world.

## Footnotes

1
http://twitter.com. Accessed January 2011.

2
http://www.epinions.com/music. Accessed August 2007.

3 See the footnote 1.

4
http://www.bing.com. Accessed January 2010.

5
http://wordlist.sourceforge.net. Accessed January 2011.

6
http://www.freebase.com. Accessed January 2011.

7
http://lucene.apache.org. Accessed January 2011.

8
http://www.cp.jku.at/CoMIRVA. Accessed January 2011.

9 The genres in C224a are Country, Folk, Jazz, Blues, R’n’B/Soul, Heavy Metal/Hard Rock, Alternative Rock/Indie Punk, Rap/Hip Hop, Electronica, Reggae, Rock’n’Roll, Pop, and Classical.

10
C224a is available at http://www.cp.jku.at/people/schedl/data/C224a.txt.

11
http://last.fm/api. Accessed January 2011.

12
http://www.allmusic.com. Accessed January 2011.

13
C3ka is available at http://www.cp.jku.at/people/schedl/data/C3ka.txt.

14
C1km is available at http://www.cp.jku.at/people/schedl/data/C1km.txt.

15
https://dev.twitter.com. Accessed February 2012.

16 Only 478 unique terms out of the 1,398 in TS_D were used, only 319 were used in at least two different tweets.

17 The red mark represents the median, the upper and lower edges of the box are respectively the 25th and 75th percentiles, the whiskers extend to the most extreme data points not considered outliers (http://www.mathworks.de/help/toolbox/stats/boxplot.html. Accessed December 2011).

18 For the experiments with alternative classifiers, similarity functions did not apply; if a classifier required a similarity function, we used WEKA’s default.
